# ASO Author Reflections: Rethinking Surgical Strategies for Pediatric Liver Tumors with Vascular Involvement: Resection with Vascular Reconstruction as an Alternative to Transplantation

**DOI:** 10.1245/s10434-025-17312-x

**Published:** 2025-04-24

**Authors:** Juri Fuchs, Sophie Branchereau

**Affiliations:** 1 Department of General, Visceral, Pediatric and Transplantation Surgery, University of Heidelberg, Heidelberg, Germany; 2 Department of Pediatric Surgery, Hôpital Kremlin-Bicêtre, APHP, University of Paris-Saclay, Paris, France

## Past

Liver tumors in children that present as large, centrally located masses involving the hepatic venous confluence or the inferior vena cava (IVC), pose significant surgical challenges.^1^ In such cases, some centers may favor liver transplantation (LT) due to the complexity of liver resection with vascular reconstruction and the risk of local recurrence.^2,3^ However, LT carries notable drawbacks, including limited organ availability, the need for lifelong immunosuppression, and potential long-term complications.^4^ Given these concerns, organ-preserving alternatives are particularly desirable in pediatric patients whenever feasible and oncologically effective. While vascular reconstruction has been investigated in adult liver surgery, its outcomes in pediatric liver tumor surgery remain unstudied. Our study aimed to assess the safety, feasibility, and oncological outcomes of extended liver resections with vascular reconstruction of the hepatic veins and/or the IVC as an alternative to LT in select cases.^5^

## Present

Our findings demonstrate that in specialized centers and with careful patient selection, major hepatectomies with hepatic vein or IVC reconstruction can be safely performed, with excellent oncologic outcomes. Among 17 children (15 hepatoblastoma, two undifferentiated embryonal sarcoma) who underwent these complex procedures, we observed no perioperative mortality, no major postoperative complications, and no local recurrences. Vascular involvement included tumor contact with all three hepatic veins or IVC infiltration. Reconstructions were performed using direct venous repair: nine cases involved the only remaining hepatic vein and eight cases involved the IVC. With an overall survival rate of 94% at a median follow-up of 44 months, our data suggest that extended liver resection can serve as a viable, organ-preserving alternative to LT in selected cases. These results highlight the importance of individualized surgical decision making and multidisciplinary expertise in pediatric liver tumor management. Our findings reinforce that pediatric liver surgery is not merely a scaled-down version of adult liver surgery, highlighting the need for pediatric-specific strategies.

## Future

Future research should focus on refining patient selection criteria to better delineate which cases are best suited for resection with vascular reconstruction versus transplantation. Additionally, collaborative multicenter studies are needed to validate our findings and further optimize surgical strategies for pediatric liver tumors with vascular involvement. The development of novel imaging techniques and intraoperative navigation tools may enhance precision in tumor resection and vascular reconstruction, further improving outcomes for children with these challenging liver tumors.
